# Observation of transverse coherent backscattering in disordered photonic structures

**DOI:** 10.1038/s41598-017-10852-7

**Published:** 2017-09-05

**Authors:** Martin Boguslawski, Sebastian Brake, Daniel Leykam, Anton S. Desyatnikov, Cornelia Denz

**Affiliations:** 1 Institut für Angewandte Physik and Center for Nonlinear Science (CeNoS), Westfälische Wilhelms-Universität Münster, 48149 Münster, Germany; 2 Nonlinear Physics Centre, Research School of Physics and Engineering, The Australian National University, Canberra, ACT 0200 Australia; 3 Division of Physics and Applied Physics, School of Physical and Mathematical Sciences, Nanyang Technological University, Singapore, 637371 Singapore; 4 School of Science and Technology, Nazarbayev University, 010000 Astana, Kazakhstan

## Abstract

Coherent backscattering, also referred to as weak localization, is an exciting multidisciplinary phenomenon that appears in disordered systems of multiple coherent-wave scattering. Providing proper scattering conditions in (2 + 1) dimensional randomized photonic systems, we optically implement, observe, and analyse transverse coherent backscattering. Ensembles of disordered wave-guide structures are prepared by random-intensity nondiffracting writing entities according to the beam’s intensity distribution. The structure size of the induced potentials naturally define an effective mobility edge, and thus, we identify a crucial impact of the plane probe waves’ spatial frequency on the strength and shape of the spectral coherent backscattering signal. We additionally observe transverse elastic scattering as a precursor of weak localization. To testify the coherent character as a fundamental condition for coherent backscattering, we propose a scheme to continuously reduce the spatial coherence of the probe beam which directly reduces the degree of localization and coherent backscattering. With our experiments, we propose a testing platform that allows comprehensive examination of coherent backscattering with a broad set of preparation parameters and under uncritical laboratory conditions. Our results are directly transferable to more complex systems of disordered wave potentials, not being restricted to photonic systems.

## Introduction

Wave scattering in a random potential under coherent conditions is naturally accompanied by coherent backscattering (CBS) and thus fosters weak localization. This is a pure wave effect and, in consequence, is ubiquitous for systems that provide multiple coherent-wave scattering. Its appearance is outlined briefly by the occurrence of particular waves for which the average probability to propagate exactly anti-parallely to the direction of incidence^1^ is significantly increased. This effect bases upon wave interference present in various systems where proper scattering conditions and sufficient long propagation distances are provided. Thus, numerous experiments were designed and realized in order to explore conditions that yield CBS, and to additionally prove the universality of this effect, e.g. by applying acoustic^2–4^, seismic^5^, as well as matter^6, 7^ or light waves in turbid colloids^8, 9^ and cold atoms^10, 11^.

Employing light as the preferred wave, a lot of efforts have been invested during the last three decades in order to analyse scattering processes inside a turbid photonic medium^12–14^, and to establish a universal theory which is consistent with experimental data^15, 16^. Since the scaling theory predicts a transition from extended to localized states in systems with dimensionality higher than two^17^, special attention has been paid to observe localization in three-dimensional samples^14^. However, from experiments on three-dimensional random media it is still challenging to distinguish whether reduced transmission rates stem from localization or from absorption and resonance^18, 19^. In particular, identifying the critical regime between localization and diffusion, the so-called mobility edge, is a burning issue still to be answered^20^.

Though all modes are localized in one- and two-dimensional random systems^17^, still, media of these dimensionalities are of high interest to investigate localization and backscattering events in various configurations as they allow for a deeper insight into scattering physics in a less complex manner^7, 21^. For there is a distinction between strong and weak localization—commonly referred to Anderson localization^22^ and coherent backscattering—, it is moreover most important to find model systems in which a transition between both regimes and comparison to ballistic propagation can be adapted at will. It has been shown in several recent works^23–26^ that proper conditions can be created in optically induced randomized potential ensembles in order to observe strong transverse localization^27^. However, conditions yielding weak localization have to be prepared carefully, as scattering might be too weak or too strong. A controllable randomness of the light potential is thus essential to detect CBS.

In this contribution we describe the preparation of ensembles of two-dimensional random photonic potential landscapes to experimentally observe and analyse CBS. To identify the impact of coherence on this effect, we moreover introduce a method to reduce the probe beam’s spatial coherence that directly implicates a decline of the observed CBS signal.

## Results

### Weak transverse scattering

**Figure 1:**
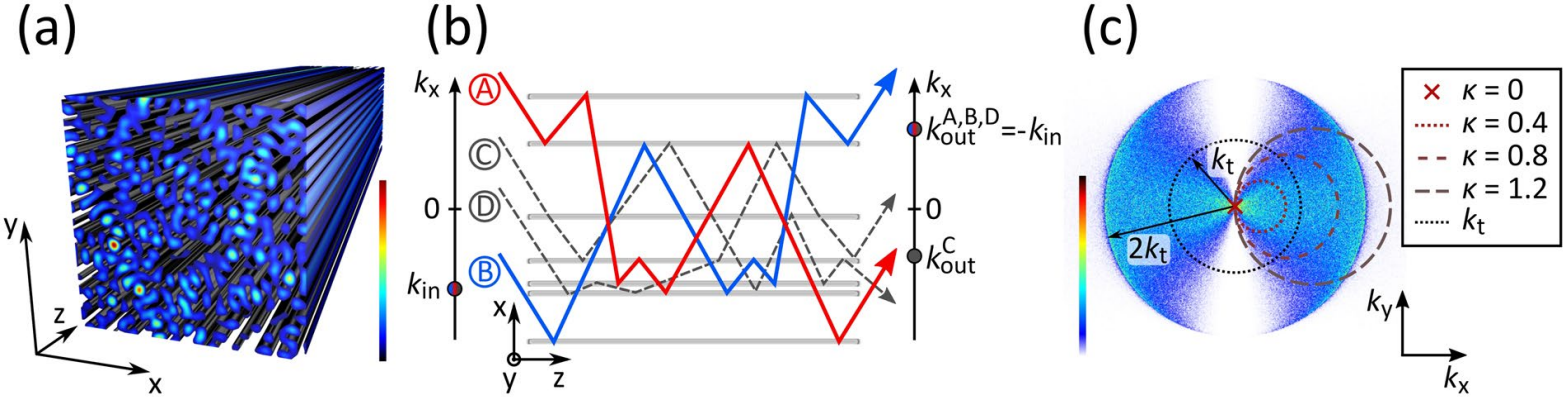
(**a**) Three-dimensional exemplification of nondiffracting random intensity distribution. (**b**) Schematic of disordered wave-guide arrangement. Gray bars represent projection of wave-guides to *x*-*z* plane. Lines indicate scattering light paths along disordered (2 + 1)-d medium. Path *B* (blue) is reversion of *A* (red), both contributing to coherent backscattering signal. Dark gray lines symbolize arbitrary light paths with random phase difference to *A* or *B*. Spatial input and output frequencies are indicated alongside. (**c**) Power spectrum of random light potential (averaged), Ewald constructions indicate probing events with various *κ*.

To observe random scattering of light, a disordered light potential is required. In our case of transverse light scattering, the disordered potential is randomly modulated in transverse plane *x*-*y* but constant in the third direction *z*, according to the schematic given in Fig. 1(a). This simple scattering scheme is illustrated in Fig. 1(b), depicting the projection of a particular (2 + 1)-d potential to the *x*-*z* plane. Scattering centres are represented by grey horizontal bars. In this scheme, four possible scattering paths of light are indicated, each with identical input wave vector ${\vec{k}}_{{\rm{in}}}$ and individual output vectors ${\vec{k}}_{{\rm{out}}}$. Since path *B* is the time inverted path of *A*, both paths have identical optical lengths. Additionally, ${\vec{k}}_{{\rm{out}}}=-{\vec{k}}_{{\rm{in}}}$ is valid, which is also the case for path *D*, yet the path length is different from *A* or *B*. The situation for two wavelets that propagate along suchlike time-reversed path pairs *A*, *B* with ${\vec{k}}_{{\rm{out}}}=-{\vec{k}}_{{\rm{in}}}$ is to necessarily interfere constructively in the far field at the spatial frequency $-{\vec{k}}_{{\rm{in}}}$. Conversely, light propagating along other paths may hold any modulus of phase difference between 0 and 2*π*. These interferences average out for extensive potential ensembles, only the contribution of these path pairs is always constructive.

In Fig. 1(c), the numerically computed power spectrum of an effective intensity resulting from the average of many random light potentials is presented. Since the potential-inducing nondiffracting beams have the spectral radius *k*_t_, the limiting spatial frequency for the potentials’ power spectra is 2*k*_t_. (See the methods section below for more information about the optical induction process in photorefractive strontium barium niobate (SBN) using nondiffracting beams.) Due to the innate orientation anisotropy in SBN crystals^28^, the power spectrum of this average light potential is deformed rather than circular symmetric as the modulation perpendicular to the *c*-axis drops to zero. This is prominent in Fig. 1(c) where many scattering centres can be found in *k*_x_ direction but no modulation is present along *k*_y_.

### Observation of coherent backscattering and elastic scattering

With a requisite of creating transversely disordered (2 + 1)-d light potentials, we establish an adequate platform to examine weak transverse localization including coherent backscattering events in transverse direction, i.e. perpendicular to the direction of light propagation. Optically induced refractive index distributions correspond to arrangements of wave-guides where randomization of the potential depth and position is introduced. Accordingly, each wave-guide is a possible scattering centre with the ability to change the incoming light field’s wave vector $\vec{k}$. Different probing scenarios are indicated in Fig. 1(c) by their respective Ewald construction for various inclinations *κ* = *k*_in_/*k*_t_.

**Figure 2:**
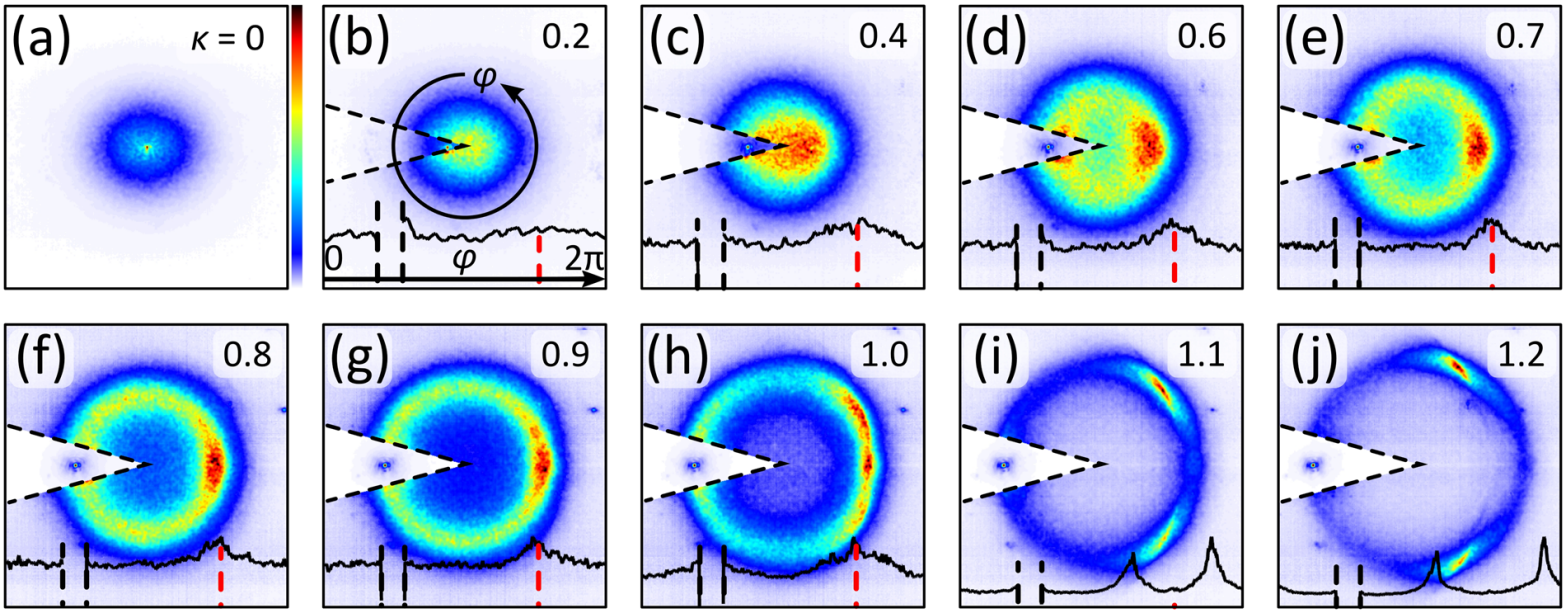
Output probability spectra ${P}_{\kappa }(\vec{k})$ for various spatial frequencies *κ*. Plots at lower part indicate azimuthal spectral distribution *P*(*φ*) at radius *k*_in_, sector of input frequency and residual sector are normalized individually. Red dashed lines mark ${\vec{k}}_{{\rm{CBS}}}=-{\vec{k}}_{{\rm{in}}}$, sector of input frequency left open.

Figure 2(a–j) show experimentally recorded probability spectra each for light propagation in an ensemble of 200 different random potentials with fixed *k*_t_. For high values of *κ*, high spectral intensities are dominant around ${\vec{k}}_{{\rm{in}}}$ indicating low scattering probability in contrast to small *κ*. In consequence, by changing ${\vec{k}}_{{\rm{in}}}$ we adjust the scattering strength of our potentials. For reasons of enhanced image dynamics and improved visibility in Fig. 2, the input sectors including ${\vec{k}}_{{\rm{in}}}$ are normalized separate to the residual spectral distribution. Plots appended to the lower part of those images represent the azimuthal spectral distribution *P*(*φ*) at radius *k*_in_, where the angle *φ* equals zero at 12 o’clock and increases counter-clockwise (cf. Fig. 2(b)). Expected CBS frequencies are marked by dashed red lines and the area around the input frequency ${\vec{k}}_{{\rm{in}}}$ is left out, again to increase the dynamics of the residual intensities.

Following the spectra from Fig. 2(a–j), we find that the characteristics of the average output spectra differ significantly. For *κ* ≤ 0.4, around perpendicular incidence, we notice an almost rotationally symmetric spectrum. Compared to a narrow discrete spectrum of the input beam, the output spectrum is broadened with maximum intensity around the origin. Accordingly, for flat-angled probing, a high ratio of light is scattered to the spectral centre. Besides the direction, also the length of the transverse wave vector is changed for this case and inelastic transverse scattering likely occurs. We verified before that for perpendicular incidence strong localization occurs^26^ for which spectral broadening is an indicator^25^.

With increasing beam tilt, enhanced spectral broadening becomes apparent where a noticeably larger amount of light is scattered to the right half of the spectrum. For 0.4 < *κ* ≤ 1, a considerable intensity peak with enhanced sharpness is found around the expected CBS frequency ${\vec{k}}_{{\rm{CBS}}}$. Here, a prominent CBS signal as an indicator of weak localization can be clearly identified. Thus, a non-zero probability is given that path pairs occur causing coherent backscattering as described above.

Additionally worth mentioning is the texture of the probability spectra apart from ${\vec{k}}_{{\rm{in}}}$ and ${\vec{k}}_{{\rm{CBS}}}$. Finding an almost filled spectrum for small tilts, the spectrum for higher *κ* values becomes more and more ring-like, referring to elastic transverse scattering. These events (rather than coherent processes) can be illustrated by applying the Ewald construction to the potential’s mean power spectrum. Along this model, all possible elastic scattering Fourier coefficients are found on a sphere of radius *k*_in_—for two dimensions the so called Ewald circle. As indicated in Fig. 1(a), its centre and the origin of the power spectrum (element of the Ewald circle) resemble the transverse components of ${\vec{k}}_{{\rm{in}}}$.

Three-dimensional elastic scattering is granted in our system as for the used low-power probe light the wave number is constant since no non-linear processes are present. However, finding transverse elastic scattering cannot be assumed directly. An explanation for its occurrence is found when considering the three-dimensional scheme of the Ewald construction. The key for this scenario is the intersection of a spectral sphere of radius *k*_in_ (the probe beam’s wave number) and the three dimensional spectrum of the light potential with ${\vec{k}}_{{\rm{in}}}$ pointing to its origin. The transverse spectral components of the light potential spectrum are depicted in Fig. 1(c). Since the potential is macroscopically elongated in *z* direction in real space, its spectrum is confined in *k*_z_ dimension, resembling a flat disc spectrum that is parallely oriented to a *k*_x_-*k*_y_ plane. In consequence, a two-dimensional Ewald construction is sufficient to model the transverse output spectrum of scattering in a light potential whose translation invariance along *z* tends to infinity. The finite elongation of the implemented potentials in our experimental case yields elastic scattering rings that are not infinitely thin but hold a particular thickness that indirectly scales with *k*_in_.

The different scenarios that are indicated in Fig. 1(c) illustrate the Ewald constructions of Fig. 2(a,c,f,j). With *κ* ≥ 1, the cut-off frequencies of the potential spectrum 2*k*_t_ are reachable. At these cut-off frequencies, the output spectrum shows substantial intensity contributions. For this case, the Ewald circle overhangs the power spectrum as its diameter is larger than 2*k*_t_ (cf. Fig. 1(c) for *κ* = 1.2). Moreover, the intersections of the Ewald circle and the cut-off frequency curve match exactly two spatial frequencies that show enhanced intensity in Fig. 2(j). Thus, at inclinations of *κ* = 1 and above we find the impact of an effective mobility edge^29, 30^.

**Figure 3:**
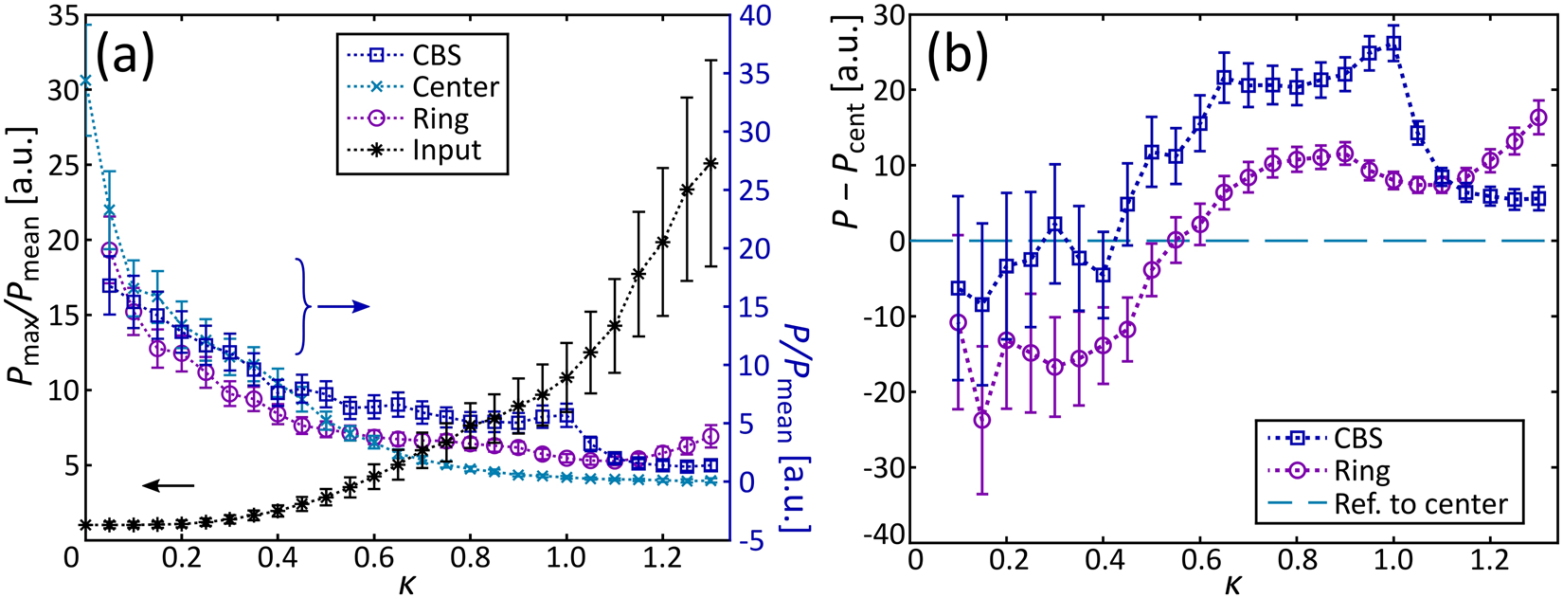
(**a**) Relative maximum *P*_max_ of output spatial frequency (black *) and spectral intensities at (blue $\square$) expected CBS frequency, (turquoise ×) at the spectral origin, (violet $\bigcirc$) at elastic scattering ring. Intensities normalized to spectral mean intensity *P*_mean_ and *P*_max_ additionally to $P({\vec{k}}_{{\rm{in}}}=\mathrm{0)}$. (**b**) Spectral intensity difference of (blue $\square$) spatial CBS frequency *P*_CBS_ and (violet $\bigcirc$) elastic-scattering ring frequency *P*_ring_ to *P*_cent_. Zero-difference intensity marked by dashed turquoise line. Errors estimated by standard error of mean intensity considering 200 realizations.

To analyse our observations more quantitatively, relative intensities of particular spatial frequencies $P(\vec{k})$ are plotted against varied tilts in Fig. 3(a). Here, $P(\vec{k})$ is normalized to the mean spectral intensity *P*_mean_. We plot the maximum of the spectrum against *κ* relating to the left ordinate and labelled in Fig. 3(a) as black asterisks. For incidence around *κ* = 0 the maximum is supposed to be found in the central area of the spectrum. Moreover, for tilts 0 < *κ* ≤ 0.3 the maximal spectral intensity is almost constant. Though, for higher tilts, the overall maximum increases and, what can be found in Fig. 2, always coincides with ${\vec{k}}_{{\rm{in}}}$. This indicates that a considerable percentage of light is transmitted rather than scattered (cf. Fig. 2(j)) which is typical for the weak-scattering regime.

Additionally, the spectral intensities at three particular spatial frequency areas are considered in Fig. 3(a), namely the expected CBS frequency with $P({\vec{k}}_{{\rm{CBS}}})={P}_{{\rm{CBS}}}$, the spectral origin $P(\vec{k}=\mathrm{0)}={P}_{{\rm{cent}}}$, and a mean spectral intensity *P*_ring_ of a *π*/16 sector at the 12 o’clock position of the elastic scattering ring. For these plots, the right ordinate is relevant. What can be found at first glance is that all considered intensities decline absolutely with increasing probe beam tilt. This is another indication for decreasing scattering probability and in agreement with our observations regarding the spectral intensity around ${\vec{k}}_{{\rm{in}}}$.

Having a closer look at the individual plots, different aspects can be identified. For small tilts, all data points represent similar values as the considered spatial frequencies *P*_CBS_, *P*_cent_, and *P*_ring_ are rather close together. As observed before, a broad output spectrum is found. With increasing tilt, intensity values split up where, among the three considered spatial frequency areas, CBS frequencies show highest values. Notice, that minimal values of *P*_cent_ together with pronounced intensities *P*_ring_ are characteristic for elastic scattering. In addition, a considerable CBS contribution is present in this regime 0.5 < *κ* ≤ 1. These characteristics are clearer observable when plotting the difference of *P*_CBS_ or *P*_ring_ against *P*_cent_, as presented in Fig. 3(b). For CBS events, a doubled spectral intensity compared to *P*_ring_ is expected^31^ and approximately found here. The uncertainty estimated by error propagation is rather high especially at lower tilts *κ*. This seems reasonable as fluctuations of high intensity values at small *κ* cause large uncertainties as discussed for Fig. 3(b).

Additionally, scattering at the effective mobility edge enhances the spectral intensity for 0.9 ≤ *κ* ≤ 1 around the CBS frequency. Above *κ* = 1, a prominent decrease of the CBS intensity is observed indicating a decreased probability of CBS path pairs to occur. This is directly connected with a lower amplitude of corresponding Fourier coefficients in the potential’s spectrum as given in Fig. 1(c). The additional effective-mobility edge contributions enhance *P*_ring_ (violet $\bigcirc$ in Fig. 3(b)) in accordance with Fig. 2(h–j).

### Suppressing weak localization

Coherent processes such as CBS cannot be explained with the help of the Ewald construction since phase effects are not considered. However, given that the observed maximum intensity at ${\overrightarrow{k}}_{{\rm{CBS}}}$ actually arises from the coherent quality of the probe beam, reducing its spatial coherence would eliminate the CBS signal.

With a probe beam of variable spatial coherence, we repeat the spectral measurements for *κ* = 0.9, as presented before, while reducing the degree of spatial coherence. (The technique for reducing the spatial coherence of a probe beam is explained in the corresponding part of the methods section). Averaging the spectral output is based on multiple-exposure recording of 100 images taken by a far-field camera.

**Figure 4:**
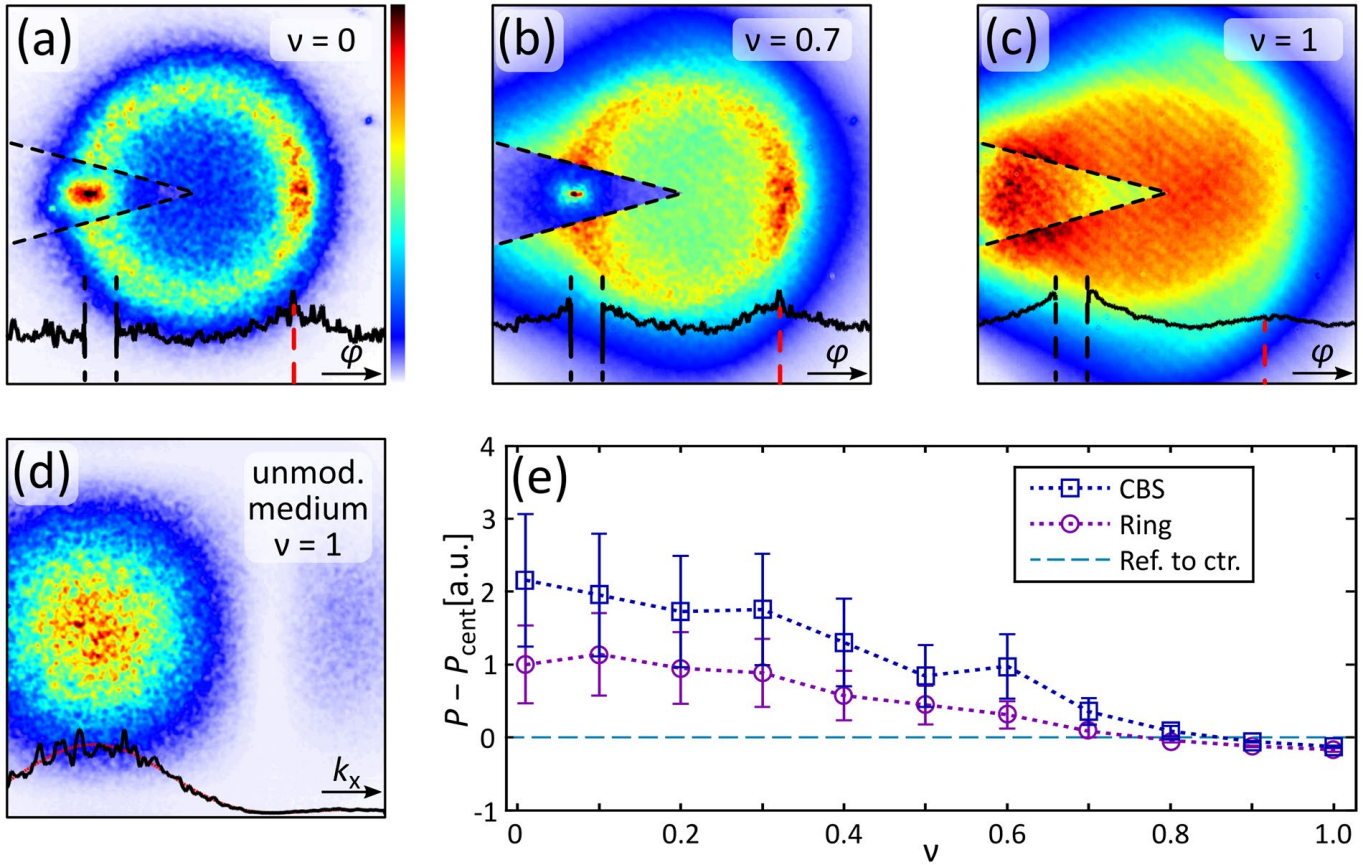
Decreasing degree of spatial coherence. (**a**–**c**) Averaged output spectra for probe-beam inclination *κ* = 0.9 and decreasing coherence, parameter *ν* denoted in upper right corner, bottom plots represent azimuthal intensity distribution with radius *k*_in_. (**d**) Spatial average spectrum of minimal spatially coherent probe beam in unmodulated medium, bottom plots represent (black) horizontal intensity through centre and (red) its sinc(*k*_x_)-fit function. (**e**) Plot of spectral intensity difference of CBS (blue $\square$) and ring frequency (violet $\bigcirc$) to central intensity (dashed turquoise line) against incoherence parameter *ν*. *P*(*ν*) normalized by *P*_ring_(*ν* = 0). Errors estimated by standard error of mean intensity considering 50 potential realizations, zero-difference intensity marked by dashed turquoise line.

The results for an altered degree of coherence are given in Fig. 4(a–c) showing the averaged output spectra for a varying degree of incoherence *ν*. As discussed before, we observe pronounced elastic scattering accompanied with a considerable CBS contribution for a probe beam of inclination *κ* = 0.9 and without phase randomization (cf. Fig. 4(a)). When increasing *ν* as indicated in Fig. 4(b) such that the random phase obviously takes effect on the spatial coherence, the central-frequency intensity is enhanced. However, the elastic scattering ring and a pronounced CBS frequency is still present.

In contrast, for a maximal phase randomization *ν* = 1 and thus the most reduced degree of spatial coherence, the characteristic of the spectrum changes drastically as shown in Fig. 4(c). That is, we find highest intensities around the centre of the spectrum. Yet, the elastic-scattering ring and a pronounced CBS signal are not present any more. Coherent backscattering is disabled by this drastic reduction of the spatial coherence. One could assume that the broad input spectrum of the randomized probe beam simply smoothens the output spectrum such that elastic scattering and CBS details merge with a blurred signal background. However, if coherence would still be present, this scenario of a blurred CBS signal would correspond to a convolution of a *ν* = 0 output spectrum (cf. Fig. 2(f)) with the spectrum of a broad Gaussian probe beam (GPB) with *ν* = 1 (cf. Fig. 4(d)). Still, a maximal value would be present around ${\vec{k}}_{{\rm{CBS}}}$. This is apparently not the case for the output spectrum depicted in Fig. 4(c) and comparable measurements.

Additional information can be extracted from Fig. 4(c). First, the boundary of the potential spectrum is visible as a faint segment in the right half outside the main contribution area. The influence of the effective mobility edge becomes more evident for this probing scheme of reduced spatial coherence. Second, one spectral part of the input beam is affected only marginally by the potentials and is found at its initial spectral position. This part is observable as a high-intensity area to the left of the randomized GPB’s central input frequency (cf. Fig. 4(a,b)).

To analyse the spectral development with reduced spatial coherence in detail, we plot differences of the spectral intensities at *P*_CBS_ and *P*_ring_ to *P*_cent_ as given in Fig. 4(e). Here, a continuous decline of both elastic scattering and CBS is present, where for *ν* ≈ 1 both spectral intensity values are almost equal and smaller than *P*_cent_. Again, as observed in Fig. 3(b), the depicted differences are accompanied by high uncertainties, especially for small *ν*.

## Discussion

In conclusion, we presented a disordered (2 + 1)-dimensional photonic wave-guide system emerging from optical induction as a platform for investigating transverse light scattering. The probed random light potentials reveal transverse elastic scattering, while the corresponding power spectrum is finite, providing an effective mobility edge^32^.

We demonstrated that, besides Anderson localization^26^, also scattering conditions are offered that lead to CBS. A variable disorder strength allows to simultaneously examine both regimes, strong and weak scattering. The transition between both regimes is given by introducing a continuous probe beam tilt. It turned out that the CBS signal is robust against a wide range of probing inclinations until the effective mobility edge takes effect and reduces the backscattering strength noticeably. However, for high inclinations, CBS can still be detected at spectral frequencies beyond the effective mobility edge where Fourier coefficients of the potential spectrum are zero.

The photonic grain size *σ* and the angle of incidence *k*_in_ turned out to be ideal control parameters for the strength of localization. We previously analysed the strength of disorder on the localization behaviour^26^, which is related to the factor kl*^33^, including the mean free path length l* assumed to be in the range of *σ*. Analysing the peak line shape of the CBS cone might be one way to identify this relation^31^. Additionally, we identified a remarkable influence on the mean output spectrum by the shape of the refractive index power spectrum with an upper limit frequency *k*_t_ which directly links to scattering behaviour transitions in the vicinity of an artificial, or effective, mobility edge^20^.

Further measurements clearly identified the essential role of spatial coherence for weak localization and CBS. We therefore decreased the spatial coherence of the probing light stepwise by introducing a continuously tunable phase randomness to the probe beam. The diminished spatial coherence fostered a continuous reduction of CBS until the signal disappeared. Our results are in accordance to an alternative method of breaking the time reversal symmetry by de-phasing pulses^34^. This approach was realized in an ultra cold-atoms system^35^ and in a multi-mode fibre^36^. However, our approach keeps the potential unaffected while only the coherence character of the probing light is altered. In reverse, a disordered silicon nano-wire system was recently examined in a backscattering configuration to identify the coherent character of spontaneous Raman scattering^37^.

Another phenomenon whose experimental implementation is highly on demand is coherent forward scattering of light^38, 39^ where our approach is a highly promising candidate to provide proper scattering conditions. With our experiments, we are convinced to pave the way for subsequent experiments and analytics that yield deeper insights into weak and strong localization phenomena.

## Methods

### Preparation of photonic random potentials

Light potentials in the shape of disordered, two-dimensional refractive index modulations are optically induced in a photorefractive strontium barium niobate crystal (SBN)^26, 40^. The particular SBN sample employed is characterised by the molecular formula Sr_0.6_Ba_0.4_Nb_2_O_6_, additionally doped with cerium. Corresponding alternative notations are SBN:Ce or SBN:60.

For inducing suchlike disordered potential configurations, we employ so-called nondiffracting random intensity patterns (NRP)^41^ as writing light fields, holding translation invariant random-intensity distributions^42^. The nondiffracting property makes these light configurations particularly appropriate to implement (2 + 1)-dimensional systems and thus to mimic the temporal development in a two-dimensional photonic configuration^43^. A three-dimensional volume is sketched in Fig. 1(a) where the trihedron indicates the orientation and direction of propagation that is congruent with the *z* direction. Selecting appropriate parameters for the structural size of such intensity—for these random beams, we recently introduced the nomenclature *photonic grain size σ* ^26^—one can reach nondiffracting distances of several centimetres and even longer. Throughout all the experiments presented here, we fix *σ* to 15 μm which is connected to a nondiffracting distance longer than the long site of the SBN crystal of dimension 5 mm × 5 mm × 20 mm. The symmetry axis (*c*-axis) corresponding to one short side is oriented parallely to the *x* direction. All writing light fields are polarized ordinarily to the *c*-axis^44^. More details about SBN which is commonly used for optical induction experiments are, for instance, given in refs 23, 28 and 43.

The backscattering character is analysed best when averaging over a large ensemble of random potentials as speckle fluctuations are averaged out. We therefore introduce an ensemble of 200 various NRP configurations for the induction of each potential with an external field of 1600 V cm^−1^ where we fix the illumination time for a single writing beam to 20 s. It turned out that this set of parameters provides a suitable refractive index modulation depth to establish appropriate scattering conditions. All given uncertainties emerge from standard error evaluations of the respective ensemble quantity and under consideration of error propagation.

We implement a set of two spatial light modulators (Holoeye Pluto in real space and LC-R 2500 in Fourier space) for the optical potential induction. Moreover, this set-up allows to linearly probe the induced potentials (cw laser light of a few s at a wavelength of *λ* = 532 nm) with a broad GPB, polarized extraordinarily to the *c*-axis^44^. Holding a beam waist of *w*_0_ = 320 μm that covers many scattering centres at the input facet, we introduce a variable tilt ${\vec{k}}_{{\rm{in}}}=({k}_{{\rm{in}}},\,0,\,\mathrm{0)}$ to the GPB along the *c*-axis direction. To circumvent the influence of the orientation anisotropy, we limit our experiments to probe beam inclinations along the direction of ${\vec{k}}_{{\rm{x}}}$. For a particular GPB tilt ${\vec{k}}_{{\rm{in}}}$, single output spectra were recorded as high-dynamic-range images by multiple exposure with a CMOS camera (uEye LE series) placed in Fourier space. We subsequently average all output spectra of induced potential realizations receiving a spectrum of probability ${P}_{\kappa }(\vec{k})$ identifying most probable spatial output frequencies. Here, we benefit from the reversibility of the SBN crystal by re-homogenizing its refractive index via illumination with a white-light LED in order to induce further structures. Typical erasure times are several seconds.

### Reduction of spatial coherence

By adding a spatially randomized phase Φ_*r*_(*x*, *y*) to the GPB, we statistically diminish its spatial coherence as the phase relation between two positions gets lost. Our method to generate Φ_*r*_ is to fragment the 2d phase distribution into tiles of 16 × 16 pixels and allocate an additional phase of *π* to randomly chosen tiles. The fill factor among 0- and *π*-phase shifted tiles is 0.5. We introduce a random phase parameter *ν* ∈ $[0,\,1]$ such that the field of a single randomized Gaussian probe beam reads as *E*_rGPB_ = *E*_GPB_ exp (*iν*Φ_*r*_(*x*, *y*)). Herein, Φ_*r*_(*x*, *y*) denotes the additional phase in the shape of randomly distributed 0- or *π*-phase tiles and *E*_GPB_ indicates the field of the unaffected GPB. The minimal degree of spatial coherence at *ν* = 1 is basically limited by the size of one phase tile.

A set of 150 probe beams are pre-calculated and randomly sent to the SLM with a refresh rate of approximately 10 Hz. A typical averaged GPB spectrum of reduced spatial coherence is shown in Fig. 4(d). Influenced by the additional random phase, the common delta-peak like spectral distribution of a plane wave turns into a broad spectrum that resembles the central maximum of a sinc(*k*_x_, *k*_y_) function. Its inverse Fourier transform is a rectangle function to be identified with the quadratic phase tiles. A sinc(*k*_x_)-fit function excellently matches the horizontal cross section depicted in the bottom part of Fig. 4(d).

### Data Availability

The datasets generated and analysed during the current study are available from the corresponding author on reasonable request.
